# The Effect of Emulsifiers on the Emulsion Stability and Extraction Efficiency of Cr(VI) Using Emulsion Liquid Membranes (ELMs) Formulated with a Green Solvent

**DOI:** 10.3390/membranes10040076

**Published:** 2020-04-21

**Authors:** Katia Anarakdim, Gemma Gutiérrez, Ángel Cambiella, Ounissa Senhadji-Kebiche, María Matos

**Affiliations:** 1 Laboratoire des Procédés Membranaires et des Techniques de Séparation et de Récupération, Département de Génie des Procédés, Université de Bejaia, Bejaia 06000, Algerie; anarakdimkatia@yahoo.fr (K.A.); kebiche_anissa@yahoo.fr (O.S.-K.); 2 Department of Chemical and Environmental Engineering, University of Oviedo, Julián Clavería 8, 33006 Oviedo, Spain; gutierrezgemma@uniovi.es (G.G.); cambiellaangel@uniovi.es (Á.C.)

**Keywords:** liquid membrane, double emulsion stability, chromium extraction, vegetable oil, PGPR

## Abstract

The stability of emulsion liquid membranes (ELMs) and their ability to extract Cr(VI) were investigated. The objective of this study is to compare different ELM formulations using combinations of two hydrophilic (Tween 20 and Tween 80) and two lipophilic (polyglycerol polyricinoleate (PGPR) and Span 80) emulsifiers. TOPO (tri-n-octylphosphine oxide) as a carrier and a green solvent (sunflower oil) were used to provide high extraction efficiency of Cr(VI). All these double emulsions were characterized in droplet size distribution, zeta potential, visual inspection with a microscope, and stability. The best formulation was obtained with PGPR as the inner stabilizer and Tween 80 as the outer stabilizer, leading to Cr(VI) ion removal efficiency of up to 96%.

## 1. Introduction

Emulsion liquid membranes (ELMs) are really double emulsions, which can be described as emulsions within emulsions. The inner aqueous phase is dispersed into the oil phase as small droplets, while the resulting simple emulsion is dispersed as big drops in the external aqueous phase. 

The structural properties of this kind of multiple emulsion lead to a high number of potential applications in medicine, pharmacy, cosmetics, and the food industries [1,2,3,4,5]. Double emulsions have also found applications in separation processes as ELMs [6,7,8,9,10,11,12,13,14,15,16]. Although this methodology has been successfully applied for chromium removal [17,18,19,20,21,22,23,24], but its commercial applications for the removal of heavy metals are limited, due to emulsion instability. The major problem regarding the stability of double emulsions is the presence of two interfaces that are thermodynamically unstable.

Preparation of a water-in-oil-in-water (W_1_/O/W_2_) emulsion requires two types of emulsifier: one with a low hydrophilic and lipophilic balance (HLB) for the W_1_/O interface, and another with a high HLB for the O/W_2_ interface. The effects of nature and concentration of both emulsifiers on double emulsions properties have been previously discussed [25,26].

**Figure 1:**
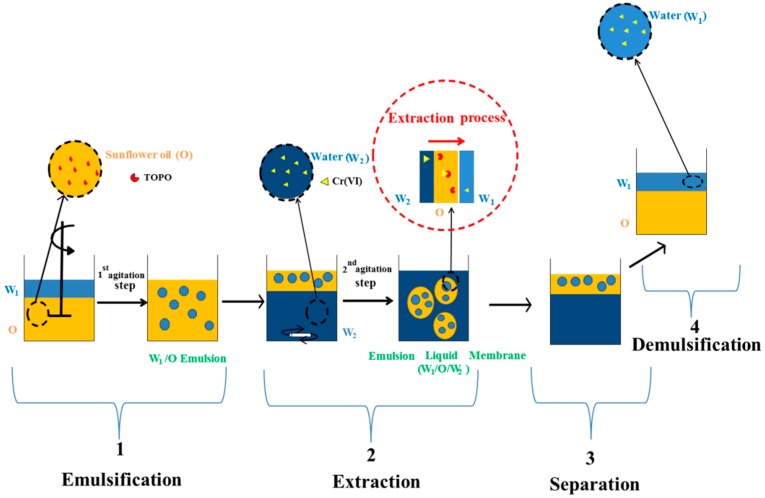
Schematic diagram for the extraction of Cr(VI) using an emulsion liquid membrane (ELM) process.

ELM processes generally consist of four steps (Figure 1). In the first step, a simple water-in-oil emulsion (W_1_/O) is prepared by mixing the membrane phase (oil phase) and the internal aqueous phase (stripping phase W_1_). The second step involves an extraction process, where the final double emulsion (W_1_/O/W_2_) or ELM is prepared by stirring the W_1_/O emulsion on the external wastewater phase to treat (W_2_). Although this double emulsion should be stable enough to allow stirring and extraction of the metal into the internal aqueous phase, it should also be easily separated after metal extraction. In the third step, the emulsion settling occurs to separate the external aqueous phase; finally, a demulsification step takes place to recover the membrane phase (oil) for its subsequent reuse.

Emulsion breakage or destabilization results in a decreasing extraction efficiency from the release of entrapped metal. One of the most important factors affecting stability is emulsion diameter. Large droplet diameters result in reduced stability and poor extraction efficiencies [6], because of a low surface/volume ratio [27]. Small droplet diameters provide more stable emulsions, a larger mass transfer area, and higher extraction efficiencies. However, if the droplet diameters are very small, the emulsion will be very difficult to destabilize by any mechanical process in the final step [6]. Therefore, in order to solve ELM stability problems, both the formulation and the preparation method should be taken into account [28,29].

The oil phase has a crucial role in ELM processes, since it is the main component of the membrane. The correct selection of the oil phase is crucial for membrane stability and for effective metal transport. As a general trend, viscous oil generally increases emulsion stability [30], but has the decreases mass transport. High density differences between the external aqueous phase (W_2_) and the oily phase would be beneficial for their separation once extraction step took place. Moreover, it is necessary that the oil phase has low water solubility to avoid interaction between both aqueous phases, which results in emulsion breakage [31]. Therefore, the most commonly oils used in ELM systems are volatile and organic solvents, such as kerosene, which has proven to work particularly well for chromium removal [10,31]. Span 80 has been used conventionally with this method as the emulsifier for stabilizing the internal aqueous phase of the liquid membrane [32]. 

It is of great interest, and a big challenge, to replace such volatile and fuel-based diluents for non-toxic vegetable oils. Soybean, palm, rapeseed, and sunflower oils have been explored in previous works: phenol removal [33,34], Cu(II) extraction [35], Cr(VI) extraction [36], the removal and recovery of rhodamine B [37], and textile dye [38]. These studies indicate a promising potential of vegetable oils in chemical extractive processes which will have an important environmental relevance. 

On the other hand, polyglycerol polyricinoleate (PGPR) is a common component in food formulations, as it is highly effective for stabilizing water/oil (W/O) emulsions [5,39,40]. Thus, it could be a good candidate to be incorporated in the formulation of EMLs for metal removal.

The purpose of this study was to optimize the formulation of ELMs in which a green solvent (sunflower oil) is selected as a diluent to provide high Cr(VI) extraction efficiency, using TOPO (tri-n-octylphosphine oxide) as a carrier. Double emulsions were formulated with combinations of two hydrophilic (Tween 20 and Tween 80) and two lipophilic (PGPR and Span 80) emulsifiers. The stability of different double emulsions was compared, and their ability to recover Cr(VI) ions was also evaluated. Aqueous droplet size distribution in W_1_/O emulsions and oil drop size distribution in W_1_/O/W_2_ double emulsions were measured, and their stability was evaluated by multiple light scattering (MLS) immediately after preparation.

## 2. Materials and Methods

### 2.1. Materials

The ELMs were formulated with four different types of emulsifiers: Tween 80 (Polyoxyethylene sorbitan monooleate) from Sigma-Aldrich (Darmstadt, Germany) with an HLB of 15.0, Span 80 (Sorbitan monooleate) from VWR International Prolabo (Radnor, PA, USA) with an HLB of 4.3, PGPR (Polyglycerol polyricinoleate) from Brenntag AG (Essen, Germany) with an HLB of 3.0, and Tween 20 (Polyoxyethylene sorbitan monolaurate) from Sigma-Aldrich (St. Louis, MO, USA) with an HLB of 16.7. The mobile carrier or extractant used was TOPO (tri-n-Octylphosphine oxide), supplied by Avocado Research Chemicals Ltd. (Morecambe, UK). Sunflower oil was used as a solvent or diluent (density = 0.689 g/cm^3^, viscosity = 0.044 Pa·s).

Analytical grade hydrochloric acid (HCl), acetone (C_3_H_6_O), sulphuric acid (H_2_SO_4_), sodium carbonate (Na_2_CO_3_·10H_2_O), and potassium chromate (K_2_CrO_4_) were purchased from Sigma-Aldrich (St. Louis, MO, USA).

### 2.2. Methods

#### 2.2.1. ELM Preparation

Cr(VI) stock solution (100 mg/L) was prepared from potassium chromate (K_2_CrO_4_). Acidic feed solutions (external aqueous phase W_2_) were prepared by adding HCl to an aqueous solution containing an appropriate amount of Cr(VI) ions. 

The sunflower oil (as diluent or green solvent), containing surfactants with different combinations (see Table 1), and TOPO (4%) previously dissolved by mechanical stirring were used as membrane phase to remove Cr(VI).

The final ELM was prepared in two emulsification steps. First, a water-in-oil emulsion (W_1_/O) was prepared by dispersing 9 mL of the stripping aqueous solution (Na_2_CO_3_·10H_2_O, 0.5 mol/L; internal aqueous phase W_1_) into 30 mL of the oil phase, using high shear mixing in an Ystral X10 mixer (Ystral GmbH, Germany), with a 6 mm dispersing tool at 5000 rpm for 20 min. Then, 10 mL of this W_1_/O emulsion was poured into 50 mL of the external aqueous phase, containing a 50 ppm of Cr(VI) at pH = 1. The system was gently stirred with an orbital agitator (400 rpm). Samples were taken from the external aqueous phase for the determination of Cr(VI) concentration by UV-Vis spectroscopy.

#### 2.2.2. Double Emulsion Characterization

Droplet size distributions were measured by laser light scattering technique in a Mastersizer S long bench apparatus (Malvern Instruments, Ltd., Malvern, UK). Samples were diluted with deionized water to prevent multiple scattering effects. The samples were then circulated through the measuring cell using a Hydro SM small volume sample dispersion unit. For the single W_1_/O emulsion, the samples were dispersed in paraffin oil (VWR International Prolabo, Radnor, PA, USA) instead of deionized water.

Three replicates were obtained for each emulsion, and results were reported as the typical droplet size distribution in µm. The mean diameters, D_[4,3]_ and D_[3,2]_, were calculated by Equations (1) and (2):

(1) $$D_{\left[4,3\right]}=\frac{\sum n_{i}d_{i}^{4}}{\sum n_{i}d_{i}^{3}}$$

(2) $$D_{\left[3,2\right]}=\frac{\sum n_{i}d_{i}^{3}}{\sum n_{i}d_{i}^{2}}$$

where $d_{i}$ is the droplet diameter, and $n_{i}$ is the number of droplets with diameter $d_{i}$. D_[4,3]_ is the volume weighted mean diameter, and D_[3,2]_ is the surface weighted mean diameter or Sauter mean diameter.

A Zetasizer Nano ZS (Malvern Instruments Ltd., Malvern, UK) was used for the zeta potential (ξ) measurements of double W_1_/O/W_2_ emulsions. Three replicates were made for each sample at of 25 °C.

Micrographs of the emulsions were obtained with a light microscope Olympus BX50 (Olympus, Tokyo, Japan) with 10–100× magnification, using UV–Vis and fluorescence lamps. 

Emulsion stability was analyzed by measuring backscattering (BS) profiles in a Turbiscan LAB apparatus (Formulaction, Toulouse, France) provided with an Ageing Station (Formulaction, France). Emulsion were placed in cylindrical glass test cells, and the backscattered light was monitored as a function of time and cell height for 4 days at the temperature of 30 °C. The sample located at the cell is optically scanned, providing BS data every 40 µm as a function of the sample height (in mm). These scans build up a fingerprint of the emulsion at a given time, providing useful information about sample stability, indicating changes in droplet size distribution or appearance of a creaming layer or a clarification front with time.

#### 2.2.3. Extraction Efficiency

The extraction efficiency, *R* (%), was calculated as

(3) $$R\left(\%\right)=\frac{{\left[\mathrm{Cr}\left(\mathrm{VI}\right)\right]}_{0}-{\left[\mathrm{Cr}\left(\mathrm{VI}\right)\right]}_{t}}{{\left[\mathrm{Cr}\left(\mathrm{VI}\right)\right]}_{0}}\times 100$$

where ${\left[\mathrm{Cr}\left(\mathrm{VI}\right)\right]}_{0}$ and ${\left[\mathrm{Cr}\left(\mathrm{VI}\right)\right]}_{t}$ are the concentrations of Cr(VI) in the external aqueous phase at time 0 and time *t*, respectively

Samples were withdrawn from the external aqueous phase after emulsion settling at the separation stage to determine Cr(VI) by UV-Vis spectroscopy (Lutterworth, UK), using 1,5-diphenylcarbazide reagent (Sigma-Aldrich, St. Louis, MO, USA) at 542 nm.

## 3. Results and Discussion 

### 3.1. Water-in-Oil (W_1_/O) Emulsions

In order to obtain high extraction efficiencies, the stability of the internal W_1_/O single emulsion must be guaranteed. This stability depends on water droplet size and the selected emulsifier, surfactant affinity for each phase (organic and aqueous) (HLB). Emulsifiers play a key role in both emulsion formation and the extraction process. The use of an appropriate emulsifier reduces interfacial tension between oil and water phases by the absorption at the liquid–liquid interface [28], ensuring the emulsion stability and enhancing the metal ion transport rate [41].

All W_1_/O emulsions were prepared at the same concentration, 4% (*v*/*v*) of lipophilic emulsifier (Span 80 or PGPR) and 1% (*v*/*v*) of hydrophilic emulsifier (Tween 80 or Tween 20), varying the combination of emulsifiers in the oil phase. Aqueous droplet size distributions in W_1_/O emulsions were measured, and their stability was also evaluated by MLS immediately after preparation. 

#### 3.1.1. W_1_/O Emulsions Stabilized with Span 80

Two combinations of Span 80 with two different hydrophilic emulsifiers (Tween 80 and Tween 20) were used. The mean diameters obtained with the Malvern Mastersizer S are given in Table 2. 

**Figure 2:**
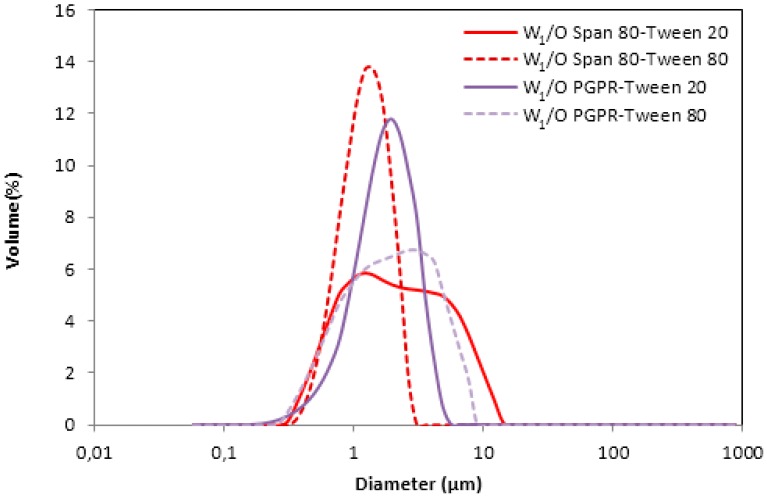
Volume droplet size distribution of W_1_/O emulsions prepared with different combinations of hydrophilic and lipophilic emulsifiers.

Figure 2 shows the volume droplet size distribution for W_1_/O emulsions stabilized with Span 80–Tween 20 and Span 80–Tween 80. For both emulsifier combinations, monomodal droplet size distribution were obtained. 

Volume-weighted mean diameter D_[4,3]_ and surface-weighted mean diameter D_[3,2]_ are presented in Table 2. It can be observed that the D_[4,3]_ value of the emulsion prepared with Span 80 and Tween 80 (1.2 µm) was significantly lower than the D_[4,3]_ value of the emulsion with Span 80–Tween 20 (2.8 µm). 

Although the D_[3,2]_ values for both emulsions were similar, the system formulated with Span 80–Tween 80 show a lower value (1.0 µm) than the system with Span 80–Tween 20 (1.4 µm).

**Figure 3:**
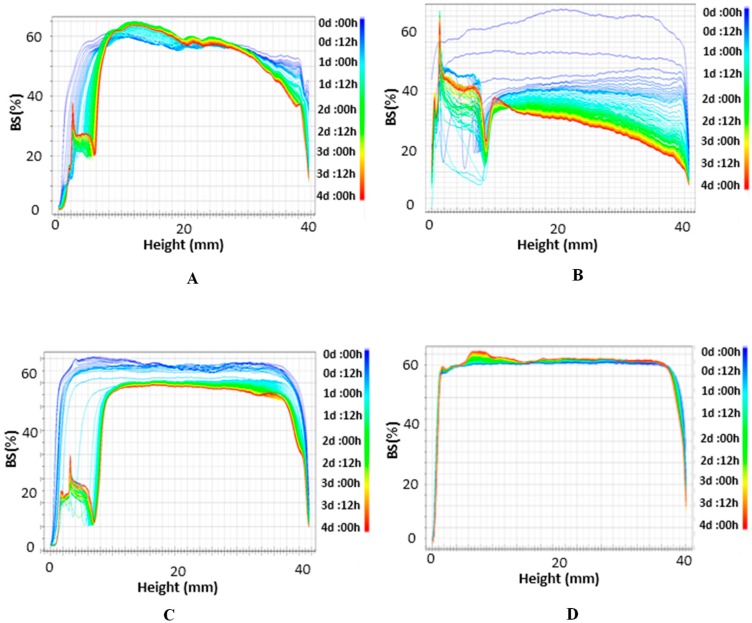
Backscattering (BS) profiles of W_1_/O emulsions stabilized with (**A**) Span 80–Tween 80, (**B**) Span 80–Tween 20, (**C**) polyglycerol polyricinoleate (PGPR)–Tween 80, and (**D**) PGPR–Tween 20.

Since D_[4,3]_ is more sensitive to the presence of small amounts of large particles than D_[3,2]_, the higher values for emulsions formulated with Span 80–Tween 20 were attributed to the presence of larger aqueous droplets. This fact could affect the stability of these emulsions, whose values are depicted by the backscattering (BS) profiles in Figure 3.

The observed decrease of the BS % values along the cells evidences an increase of aqueous drops size probably due to a coalescence process. Moreover, the concurrent increase of BS % at the bottom of the cell and reduction at the top indicates that a sedimentation process of the aqueous droplets was taking place. 

Thus, coalescence and sedimentation were accelerated in emulsions formulated with Span 80–Tween 20 (Figure 3B). In contrast, no considerable BS variations were observed in the middle part of the cell for systems prepared with Span 80–Tween 80; the variation was ~4% in the first two hours, which means that there were no substantial changes in droplet size, so the emulsion remained stable (Figure 3A). After this time, an increase of BS variations in the middle part of the cell indicates that coalescence is starting to take place.

The different stabilization behaviour of these emulsions could be attributed to (a) the compatibility of Tween 80 and Span 80, or (b) the lower HLB value of the combination Span 80–Tween 80 with respect to Span 80–Tween 20, which is what makes them more appropriate to formulate W_1_/O emulsions.

#### 3.1.2. W_1_/O Emulsions Stabilized with PGPR 

Table 2 shows the mean diameters provided by the Malvern Mastersizer S for the two combinations of PGPR with the hydrophilic emulsifiers (Tween 80 and Tween 20). The same D_[3,2]_ value (1.3 µm) was obtained for both W_1_/O emulsions. Nevertheless, the D_[4,3]_ value of the emulsion stabilized with PGPR–Tween 80 (2.3 µm) was significantly higher than the D_[4,3]_ value of the emulsion with PGPR–Tween 20 (1.8 µm).

Volume droplet size distributions for W_1_/O droplets stabilized with PGPR at different combination of hydrophilic emulsifiers are given in Figure 2. In all cases, the emulsions again showed monomodal droplet size distributions. 

BS profiles of the primary W_1_/O emulsions formulated with PGPR are also included in Figure 3. For the emulsion stabilized with PGPR–Tween 80 (Figure 3C), the decrease of the BS % values along the cell indicates an increase of aqueous drops size due to a presumable coalescence process. Furthermore, the increase of BS % at the bottom of the cell with the simultaneous decrease of BS % at the top indicates a sedimentation of the aqueous drops. For the emulsions formulated with PGPR–Tween 20, no clear front or BS variations were observed (Figure 3D), which is an indication that this emulsion was stable with time, at least during the measuring time (4 days).

For the emulsion stabilized with PGPR, the most effective emulsifier was Tween 20. However, when Span 80 was used as stabilizer, the most effective emulsifier was Tween 80.

Equation (4) was used to calculate the HLB of the different systems (HLB_mixture_; results shown in Table 3):

HLB_mixture_ = (W_A_ × HLB_A_) + (W_B_ × HLB_B_) (4)

where W_A_ and W_B_ are the weight fraction of each emulsifier, and HLB_A_ and HLB_B_ are the HLB values for each emulsifier, respectively.

Taking into account the HLB_mixture_ values, it can be concluded that the stability of the emulsion is not solely dependent on the HLB of the emulsifier. If that were the case, both systems formulated with PGPR should show high stability, but that only happens for the system stabilized with PGPR–Tween 20, whose HLB value is only slightly higher than the one shown by the PGPR–Tween 80 system. 

Therefore, the differences found in emulsion stability should be attributed to the chemical compatibility of emulsifiers concerned, and probably due to the presence of a double bound in the hydrocarbon chain of Tween 80 and Span 80, which decreases their hydrophobicity making them less suitable to stabilize W/O emulsions [42].

### 3.2. Water-in-Oil-in-Water Emulsions (W_1_/O/W_2_) 

W_1_/O/W_2_ double emulsions (ELMs) were prepared with different combinations of emulsifiers, in order to select the formulation that provides good phase dispersion and leads to high metal extraction efficiency.

**Figure 4:**
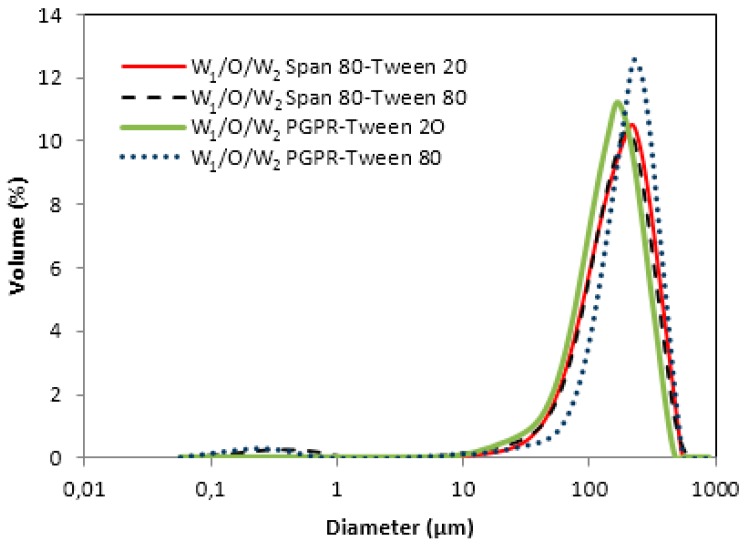
Volume droplet size distribution of water-in-oil-in-water (W_1_/O/W_2_) double emulsions prepared with different combinations of hydrophilic and lipophilic emulsifiers.

Figure 4 shows monomodal droplet size distributions for all the emulsions formulated. Large drops from 100 to 200 µm were obtained, with one well-defined peak at 193 µm and another at 150 µm for emulsions formulated with PGPR–Tween 80 and PGPR–Tween 20, respectively. For W_1_/O/W_2_ double emulsions formulated with Span 80, drop sizes ranged from 160 to 180 µm for both emulsifiers. 

The main droplet diameters D_[3,2]_ and D_[4,3]_ are given in Table 4. 

When the lipophilic emulsifiers (Span 80 or PGPR) were combined with Tween 20, larger drops (117 µm and 100 µm, respectively) were obtained. On the contrary, when W_1_/O/W_2_ double emulsions were formulated with Tween 80 as the emulsifier, small droplets were produced, leading to sizes of 6.8 µm for Span 80 and 4.9 µm for PGPR. This fact indicates that W_1_/O/W_2_ double emulsions were stabilized by the combination of PGPR, and that Tween 80 will be more stable.

The mean zeta potentials values are also listed in Table 4. Low zeta potentials at the O/W_2_ interface facilitate the aggregation of oil drops, but the non-ionic character of the emulsifiers explains all these low values. The emulsion formulated with the combination of PGPR–Tween 80 showed the lowest zeta potential value, which was very close to zero.

**Figure 5:**
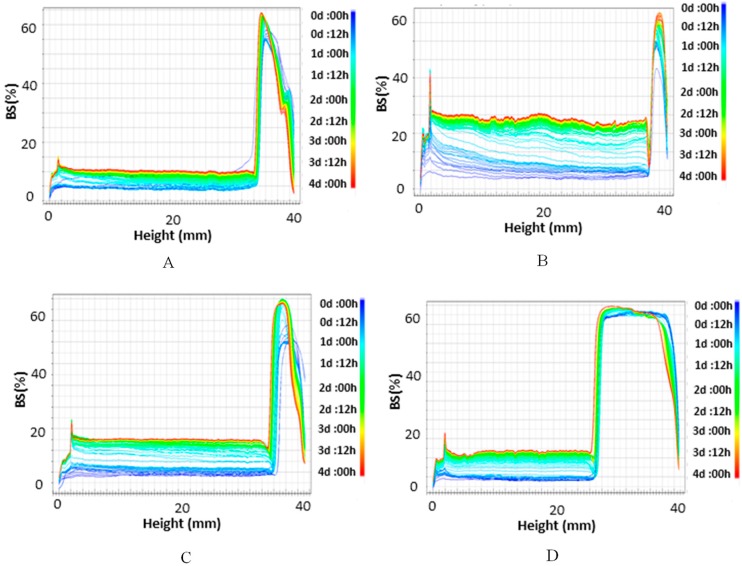
Backscattering (BS) profiles of W_1_/O/W_2_ double emulsions stabilized with (**A**) Span 80–Tween 80, (**B**) Span 80–Tween 20, (**C**) PGPR–Tween 80, and (**D**) PGPR–Tween 20.

BS profiles for W_1_/O/W_2_ double emulsions with all combination of emulsifiers are given in Figure 5. 

BS profiles for the emulsions stabilized with PGPR (Figure 5A,B) and Span 80 (Figure 5C,D) show two regions that can be clearly distinguished. A clarification process is observed along the height of the cell, with a decrease in BS at the bottom and an increase on the top, due to the lower density of oil drops, which rise towards the top of the cell, leading to a creaming process. Moreover, BS variations are also caused by oil drop coalescence.

**Figure 6:**
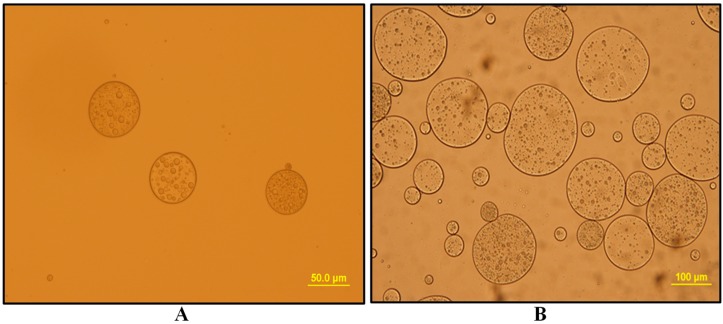

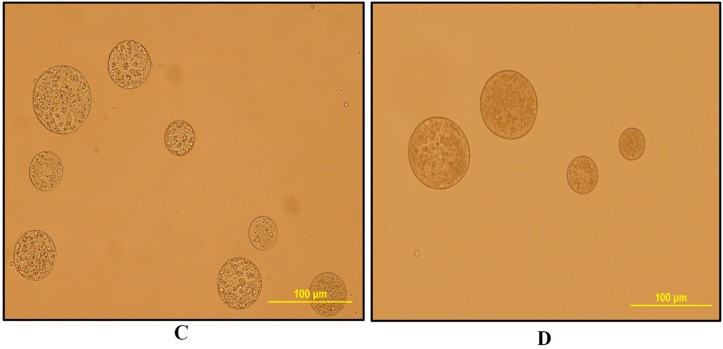
Optical microscopy images of W_1_/O/W_2_ double emulsions stabilized with (**A**) Span 80–Tween 80, (**B**) Span 80–Tween 20, (**C**) PGPR–Tween 80, and (**D**) PGPR–Tween 20.

Oil drops containing small aqueous droplets can be clearly observed in Figure 6, ensuring the presence of double emulsions. 

Visual inspection of emulions micrographs indicates that only the emulsion prepared with Span 80–Tween 20 (Figure 6B) was not stable, since the oil drops quickly lost their spherical shape and coalesced.

### 3.3. Effect of the Surfactants on the Extraction Efficiency of Cr(VI)

Emulsifiers added to ELM systems influence not only the stability of the double emulsions, but also the mass transfer resistance (extraction efficiency). This finding is consistent with previous works [43], where it was found that the selection of the surfactant is a key factor to reduce emulsion swelling and hence membrane breakage, enhancing extraction efficiency.

**Figure 7:**
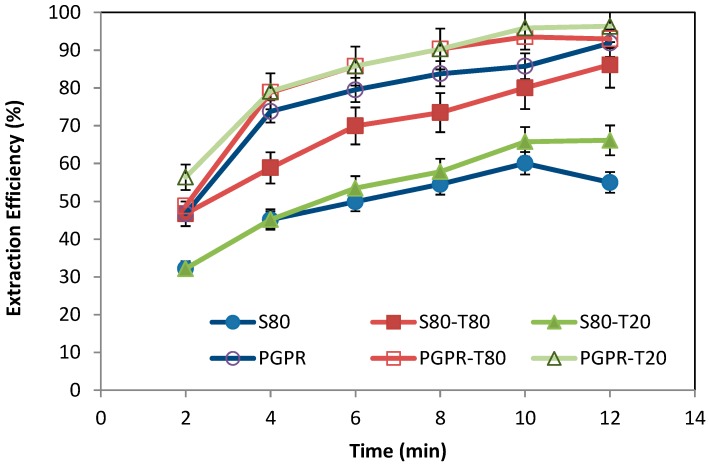
Effect of the type of emulsifiers as emulsion stabilizer on the Cr(IV) ions extraction efficiency over time. S80: Span 80; PGPR: polyglycerol polyricinoleate; S80-T80: Span 80–Tween 80; S80-T20: Span 80–Tween 20; PGPR-T80: PGPR–Tween 80; PGPR-T20: PGPR–Tween 20.

The effect of lipophilic emulsifiers alone and the influence of different combinations with hydrophilic emulsifiers on the removal efficiency of Cr(VI) ions with time is shown in Figure 7. 

As usual, extraction efficiency increases with contact time in all cases. PGPR shows higher efficiency values than Span 80, which could be attributable to the higher stability of the W_1_/O emulsions formulated with PGPR as the emulsifier. Wan and co-workers [43] also observed that the use of polymeric surfactant (lauryl methacrylate, LMA), with high molecular weight and low hydration capability, forms the stable ELMs with reduced emulsion swelling compared with other commercial surfactants such as Span 80.

The addition of the hydrophilic emulsifiers (Tween 80 and Tween 20) has allowed an improvement and increase in extraction efficiency with both different lipophilic emulsifiers. Björkegren and co-worker [44] suggested that hydrophilic surfactant Tween 80 reduces emulsion viscosity, and enables the creation of a double emulsion during the extraction process. Hydrophilic emulsifiers stabilize the double emulsion, resulting in a homogeneous solution when the three phases are placed together during extraction process, hence producing a positive effect on the overall chromium extraction efficiency.

In the extraction step, stirring to disperse the emulsion phase in a continuous feed phase was done. The emulsion should be stable enough against stirring during extraction hexavalent chromium process, which means that more emulsion is stable and therefore the extraction efficiency will be better. The results of the extraction study with regard to the stability and the characterization of the emulsions revealed a significant correlation between stability and the extraction efficiency: the emulsions prepared with PGPR–Tween 80 wand the one prepared with PGPR-Twee 20 present high stability and gave the best extraction efficiency of 96.33% and 93.98% respectively. The lowest extraction efficiency value was obtained when Span 80 and Tween 20 were used as stabilizers, leading an extraction efficiency of 66.2%. These results can be explained because of the large size of the inner oil drops, which leads to their easier coalescence, as was confirmed by visual inspection (Figure 6B). Therefore, there is a decrease of interfacial area, and hence the mass transfer rate. Ahmad and co-workers [8] found that the removal capacity of cadmium was a function of emulsion diameter, and so it has been seen that removal capacity decreases with the increase of the diameters of droplets. 

All values seem to reach a plateau, indicating the saturation of the inner phase on the metal extracted, indicating the maximum concentration possible by the systems. The maximum concentration was observed to be different for each system, since significant differences are observed for extraction efficiency.

## 4. Conclusions 

It has been proved that ELMs can be formulated using a vegetable oil as a green solvent while maintaining high Cr(VI) extraction efficiency, if appropriate stabilizers are used.

ELMs formulated with Span 80 as an inner lipophilic emulsifier, with the both hydrophilic emulsifiers (Tween 20 and Tween 80) and sunflower oil as solvent, resulted in unstable W_1_/O emulsions with low recovery of Cr(VI). Moreover, oil-phase separation after the extraction step became difficult due to a third phase formation.

Better results were obtained when sunflower oil was used in combination with PGPR as an inner phase emulsifier, yielding extraction efficiencies of up to 96%. The best formulation was the combination of PGPR with Tween 80 as an outer emulsifier, since it provided not only good stability and high extraction efficiency of Cr(VI) at the extraction step, but also adequate performance in the subsequent separation and demulsification steps. 

Extraction efficiencies raise the plateau in all cases, probably due to the maximum concentration that the system can extract; this maximum concentration differs from one system to another. The concentration of Cr(VI) in the final stripping aqueous phase produce values in the range of 1200–2100 mg/L for all systems formulated.

## Figures and Tables

**Table 1:** Mean droplet diameters of water-in-oil (W_1_/O) emulsions prepared with different combinations of hydrophilic and lipophilic emulsifiers.

Lipophilic	Hydrophilic	D_[3,2]_ (µm)	D_[4,3]_ (µm)
Span 80	Tween 80	1.0	1.2
	Tween 20	1.4	2.8
PGPR	Tween 80	1.3	2.3
	Tween 20	1.3	1.8

**Table 2:** Different combinations of surfactants.

	Different Combinations of Surfactants
1	4% (*v*/*v*) Span 80 and 1% (*v*/*v*) Tween 80
2	4% (*v*/*v*) Span 80 and 1% (*v*/*v*) Tween 20
3	4% (*v*/*v*) PGPR and 1% (*v*/*v*) Tween 80
4	4% (*v*/*v*) PGPR and 1% (*v*/*v*) Tween 20

**Table 3:** Hydrophilic and lipophilic balance (HLB) values for mixtures of different emulsifiers (HLB_mixture_).

HLB_mixture_			
Span 80–Tween 80	Span 80–Tween 20	PGPR–Tween 80	PGPR–Tween 20
6.4	6.8	5.4	5.7

**Table 4:** Mean droplet diameters and zeta potential values of W_1_/O/W_2_ double emulsions prepared with different combinations of hydrophilic and lipophilic emulsifiers.

Lipophilic	Hydrophilic	D_[3,2]_ (µm)	D_[4,3]_ (µm)	Zeta Potential (mV)
Span 80	Tween 80	6.76	167	5.1 ± 2.3
	Tween 20	117	179	1.3 ± 2.1
PGPR	Tween 80	4.92	193	−0.2 ± 3.5
	Tween 20	100	150	4.6 ± 2.0
